# Artificial Intelligence in U.S. Surgical Training: A Scoping Review Mapping Current Applications and Identifying Gaps for Future Research Applications

**DOI:** 10.7759/cureus.93793

**Published:** 2025-10-03

**Authors:** Yasmine Zerrouki, Jessica V Baran, Rishiraj Bandi, Elijah Moothedan, Michelle K Knecht, Tiffany Follin, Parvathi Perumareddi

**Affiliations:** 1 Department of Medicine, Florida Atlantic University Charles E. Schmidt College of Medicine, Boca Raton, USA; 2 Department of General Surgery, University of South Florida Morsani College of Medicine, Tampa, USA

**Keywords:** artificial intelligence (ai), general surgery residency training, machine learning (ml), medical resident education, teaching technology

## Abstract

Artificial intelligence (AI) and machine learning (ML) are increasingly applied in general surgery education, yet their full impact on training across different learner levels remains unclear. The objectives of this study are to map the current use of AI/ML in general surgery education, with a focus on skill acquisition, risk stratification, and competency evaluation. The eligibility criteria included studies involving AI/ML interventions in general surgery training for medical students, residents, or practicing surgeons. Articles focused solely on clinical outcomes or non-surgical fields were excluded. A systematic search of databases, including PubMed, Scopus, Embase, and IEEE Xplore, was conducted. Data were extracted on study design, training level, type of AI/ML tool, educational focus, and key outcomes. A total of 18 studies were selected, which focused on simulation-based training, skill assessment, and decision support. Common barriers included a lack of standardization and limited integration into curricula. AI/ML shows promise in enhancing surgical education, but further research is needed to validate tools, measure impact, and address integration challenges.

## Introduction and background

Artificial intelligence (AI) has been recently integrated into medical education, research, and clinical practice for both educational and clinical purposes [1-3]. The scope of its application covers diagnostic assessments to personalized patient care plans to identification of complications, often utilizing predictive analytics and sophisticated algorithms [4]. Notably, AI has been incorporated in the fields of radiology and oncology by optimizing diagnostic and imaging accuracy for improved patient outcomes [5,6].

General surgery, a field known for its technical demands and high-stakes decision-making, presents unique opportunities for integrating AI into training and practice [7]. Surgical education spans a continuum from medical students learning foundational skills, to residents mastering surgical techniques, to fellows and attending surgeons refining advanced procedures and assessing risks [8]. Given the importance of competency-based assessments, risk evaluation, and manual dexterity, general surgery serves as an ideal domain for exploring AI’s potential to enhance training outcomes [9].

AI applications in general surgery education include risk stratification, where algorithms assist trainees in understanding and mitigating patient-specific risks; simulation-based training, where machine learning (ML) models provide personalized feedback on manual skills; and decision-support tools that guide clinical judgment [10]. These innovations show promise in improving the accuracy of assessments, reducing training variability, and accelerating the development of surgical expertise [7].

This scoping review aims to map the current use of AI and ML across the continuum of general surgery training, from medical students to practicing surgeons, and to identify barriers to their implementation. While prior studies have explored specific applications of AI in surgical practice, there remains a lack of comprehensive synthesis on how these technologies are being integrated into surgical education and training. Given the rapidly evolving nature of AI and the diverse ways it may influence skill acquisition, risk stratification, and competency evaluation, a scoping review is well-suited to capture the breadth of existing evidence, highlight patterns of use, and identify gaps in knowledge to guide future research and curriculum development.

## Review

Methodology

The Preferred Reporting Items for Systematic Reviews and Meta-Analyses Extension for Scoping Reviews (PRISMA-ScR) was utilized as a reference checklist for this study. The Arksey and O’Malley Framework (2005) was used to guide the methodology for this review [11]. This includes the five-step structure of (1) identifying research questions; (2) searching for relevant articles; (3) extracting data and summarizing studies relevant to the research questions; (4) charting the data; and (5) collating, summarizing, and reporting results. The Joanna Briggs Institute recommendations were used for the extraction, analysis, and presentation of results for the review. The search strategy was peer-reviewed by TF, a research librarian.

Step 1. Identifying Research Questions

Three guiding research questions were used for this scoping review: (1) What are the current trends, challenges, and opportunities in the integration of AI and ML technologies within surgical training?; (2) What future applications of AI and ML technologies in surgical training are being explored, and what are the positive and negative consequences of these?; (3) How do these advancements impact patient outcomes, surgery residency diagnostic acumen, and clinical decision-making?

Step 2. Searching for Relevant Articles

Keywords and search terms were developed in collaboration with TF, a research librarian. Search terms addressed the following: Healthcare; Machine Learning; Artificial Intelligence; Risk Assessment; Surgical Practice; Surgery; Cancer; Bowel Surgery; Thyroid Surgery; Malignancy; Cancer screening; Disease progression. Searches were performed in four databases (PubMed, Embase, Scopus, and Cochrane Library), and a review of the literature was completed over four months, from July 2024 to October 2024, starting on June 13, 2024. Screening of the articles based on our inclusion and exclusion criteria was carried out by the coauthors YZ, JB, EM, and RB. To limit biases throughout the screening process, initial screening of relevant articles was conducted by four co-authors in two groups of two (RB and EM; YZ and JB). A second screening of the articles was performed by the senior author for a final decision on the total number of articles to include in this scoping review. The search protocol implemented for this review has not been formally registered through any official registries.

Inclusion Criteria

The scoping review included peer-reviewed qualitative, observational, or experimental studies published between 2014 and 2024 that explored the current use of AI and/or ML in general surgery surgical training. These studies must have involved medical students, medical residents, and/or attending surgeons to focus on the benefit of AI specific to the scope of these healthcare professionals. The papers examined patient post-surgical outcomes following the use of machine learning and AI to determine risk, predict disease progression, and manage patients with early stages of disease before surgical intervention, and determine the need for surgery. Included studies also examined the use of ML to assess surgical training skills and patient post-surgical outcomes following the use of ML and AI. Studies must have involved patients and participants greater than 18 years old. All included studies were based in the United States due to the specific structure and expectations of general surgery training compared to other nations.

Exclusion Criteria

Excluded studies included published abstracts, narrative reviews, scoping reviews, systematic reviews, and editorials. Studies that did not focus on general surgery or general surgery subspecialty training were excluded, such as articles examining plastic surgery, urology, neurosurgery, or orthopedic surgery training, to limit the scope of this review. Studies performed outside of the United States, before 2014, and on patients under the age of 18 were also excluded due to the ever-evolving nature of AI and the goal to keep this study as up to date and applicable to new trends in AI as possible.

Step 3. Data Extraction and Summarization of Studies Relevant to the Research Questions

All co-authors (YZ, RB, JB, EM) extracted data and summarized data relevant to the research questions. The senior author (PP) reviewed all tabulated data to resolve any discrepancies. Summary tables included an evidence table (Table 1) with tabulated study characteristics. Table 2 lists the lessons learned within the selected studies. Table 3 includes the major findings and future recommendations identified in the relevant studies. Basic qualitative content analysis was performed to identify similar themes in future directions and recommendations across studies highlighted in Table 3.

**Table 1:** General characteristics of selected articles.

Primary author/Year	Study design	Sample size	Study population	Study purpose	Major findings	AI/ML technique or program name
Azari et al., 2019 [12]	Observational study by machine learning programs	N = 37 participants (10 medical students, 15 residents, 10 attending physicians, and 2 retired physicians)	All participants were approved by the University of Wisconsin-Madison	“This study explores how common machine learning techniques can predict surgical maneuvers from a continuous video record of surgical benchtop simulations”	“Random forest predictions of surgical video correctly classified 74% of all video segments into suturing, tying, and transition states for a randomly selected test set. Hidden Markov model adjustments improved the random forest predictions to 79% for simple interrupted suturing on a subset of randomly selected participants”	Decision trees, Random forests, Hidden Markov models
Brennan et al., 2019 [13]	Prospective non-randomized study by a novel machine learning program	N = 20 participants (attending physicians and residents)	All participants were selected from a single academic quaternary care institution affiliated with the University of Florida	“Data-driven, predictive risk algorithms such as MySurgeryRisk implemented in an intelligent decision support platform can simplify and augment physicians’ risk assessment”	“The Area Under the Curve of the MySurgeryRisk algorithm was significantly better than physicians’ initial risk-assessments for all postoperative complications except cardiovascular. After interaction with the algorithm, the physicians significantly improved their risk-assessment for acute kidney injury and for an intensive care unit admission greater than 48 hours. Physicians rated the algorithm easy to use and useful”	MySurgeryRisk
Chapman et al., 2017 [14]	Data analysis using natural language processing methods	665 documents of electronic health records for 505 patients	Patients undergoing gastrointestinal surgery from the MIMIC-III Critical Care Database	Natural Language Processing methods can be used to efficiently extract information from Electronic Health Records to diagnose surgical site infections	fcFinder and fcClassifier identify surgical site infections in a much more effective and faster timeline while having the same accuracy as manual chart review	Fluid Collection Finder, pyConText
Corey et al., 2018 [15]	Single-site study with retrospective data	66,370 patients who had undergone 99,755 invasive procedural encounters in total	“Patients who underwent invasive procedural encounters between January 1, 2014, and January 31, 2017”	“In an effort to better identify high-risk surgical patients from complex data, a machine learning project trained on Pythia was built to predict postoperative complication risk”	“Compared to heuristics that identify high-risk patients developed by clinical experts and the ACS NSQIP calculator, this tool performed superiorly, providing an improved approach for clinicians to estimate postoperative risk for patients”	Least Absolute Shrinkage and Selection Operator
El Chaar et al., 2019 [16]	Retrospective cohort study	101,599 patient cases	2015 Metabolic and Bariatric Surgery Accreditation Quality Improvement Project (MBSAQIP) Participant User Data File Patient List	“This retrospective study used the 2015 Metabolic and Bariatric Surgery Accreditation Quality Improvement Project (MBSAQIP) database to evaluate patient outcomes for gastric bypass (GB) and sleeve gastrectomy and to develop a risk prediction model for serious adverse events(SAEs) and readmission rates 30 days after surgery”	“Our exploratory regression models may be used by clinicians to counsel patients about surgical risks, although future external validation should occur in non-North American populations”	N/A
Hassan et al., 2015 [17]	Randomized prospective crossover study	40 subjects (32 medical students and 8 junior residents)	Participants were taken from the general surgery program at St. Agnes Hospital	“Robotic training (RT) using the da Vinci skills simulator and conventional training (CT) using a laparoscopic ‘training box’ are both used to augment operative skills in minimally invasive surgery. The current study tests the hypothesis that skill acquisition is more rapid using RT than using CT among naive learners”	“Speeds were faster overall with RT than with CT, but the percentage of speed improvement with trials was similar, suggesting similar learning curves, with minimal transfer effect appreciated”	N/A
Hernandez et al., 2014 [18]	Retrospective cohort study	156 patients, reviewed by 1 surgeon	Tertiary referral center affiliated with the USF College of Medicine	“To use regret decision theory methodology to assess three treatment strategies in pancreatic adenocarcinoma”	“Compared with the ‘always aggressive’ or ‘always passive’ surgical treatment strategies, the survival model was associated with the least amount of regret for a wide range of threshold probabilities”	Cox Proportional Hazards Model
Kanitra et al., 2021 [19]	Randomized control trial	n = 40	Medical students at Michigan State University	“To assess the performance of novice medical students in robotic compared to laparoscopic surgery, transference of pre-existing skills between the two modalities, and to measure the learning curves. Additionally, we compared the mental and physical implications of the two modalities”	“Students who first underwent training in LS performed better in RS than students who started out in RS, suggesting a transference of skills from laparoscopic to robotic surgery”	N/A
Kulkarni et al., 2022 [20]	Randomized control trial	n = 13	Medical students at the Virginia Tech School of Medicine	“To establish the sensitivity and relative importance of different scene-dependent gaze and motion metrics for estimating trainee proficiency levels in surgical skills”	“This research presents a CV-based method to compute a set of scene-dependent eye gaze metrics that are useful for identifying proficiency levels and are sensitive in differentiating proficiency levels; the clustering analysis of the six metrics were able to identify three proficiency levels that reflect progression of skill acquisition, extending beyond differentiation between experts and novices that serves as typical criterion of metric sensitivity and validity in prior work”	Random Forest Model
Namazi et al., 2023 [21]	Data analysis using ML model	N/A	N/A	“To propose a novel context-aware model called LapTool-Net to detect the presence of surgical instruments in laparoscopic videos”	“The results show LapTool-Net outperformed state-of-the-art methods significantly, even while using fewer training samples and a shallower architecture. Our context-aware model does not require an expert's domain-specific knowledge, and the simple architecture can potentially improve all existing methods”	Lap-Tool Net
Parreco et al., 2018 [22]	Data analysis using Natural Language Processing methods	n = 3,838	SICU Admissions at Beth Israel Deaconess Medical Center from 2001 to 2012	“To use natural language processing of physician documentation to predict mortality in patients admitted to the surgical intensive care unit (SICU)”	“This study demonstrates the novel use of artificial intelligence to process physician documentation to predict mortality in the SICU. The classifiers were able to detect the subtle nuances in physician vernacular that predict mortality. These nuances provided improved performance in predicting mortality over physiologic parameters alone”	Natural Language Processing
Perumalla et al., 2023 [23]	Qualitative analysis	n = 52	Attending and retired surgeons at the American College of Surgeons Clinical Congress in San Francisco	“To develop and evaluate an algorithm to identify basic maneuvers such as suturing, knot tying, and cutting”	“We have demonstrated that a reasonably accurate AI-enabled method to automatically detect basic maneuvers can be developed and tested on a data set of simulated enterotomy repair videos. A qualitative concept mapping was demonstrated to show that real-life scenarios can benefit from the identification of basic maneuvers”	ResNet-50, Long-Short Term Memory Unit
Smith et al., 2022 [24]	Data analysis using deep neural networks	254 sessions	Surgeons at Advent Health Nicholson Center, CA	“We propose that modern, advanced machine learning methods, specifically deep neural networks (DNN), could be trained to provide a score that captures performance with the consistency of computer simulation algorithms and the holistic evaluation of a human instructor”	The DNN models matched the classifications applied by human evaluators with 83.1% accuracy for the Ring & Rail exercise and 80.8% for the Suture Sponge exercise	Deep Neural Network (DNN)
Watson, 2014 [25]	Prospective Observational Study by Machine Learning Programs	n=24	University of North Carolina at Chapel Hill, attending surgeons and surgical residents in their first and second postgraduate years	To test a machine learning algorithm to classify the expertise of a learner performing a specific surgical task	Support vector machine (SVM) algorithm increased the predictive power to classify blinded surgical hand motion patterns into expert versus novice groups. The SVM algorithm had an accuracy of 83% (sensitivity 86%, specificity 80%)	Support Vector Machine Classification Algorithm
Winkler-Schwartz et al., 2019 [26]	Prospective observational study by machine learning programs	n = 50	14 surgeons, 4 fellows, 10 senior residents, 10 junior residents, and 12 medical students from a single university	To identify surgical and operative factors selected by a machine learning algorithm to accurately classify participants by level of expertise in a virtual reality surgical procedure	The machine learning algorithm successfully classified participants into four levels of expertise with 90% accuracy with as few as six performance metrics	K-nearest neighbor algorithm, Naive Bayes algorithm, Discriminant Analysis Algorithm, Support Vector Machine Algorithm
Wu et al., 2021 [27]	Prospective observational study by machine learning programs	n = 7	Seven surgical trainees (residents and medical students) in urology who had no formal robotic training experience from a large academic medical school	To measure changes in trainees’ cognitive and behavioral states as they progressed in a robotic surgeon training curriculum at a medical institution	Changes in performance were negatively correlated with changes in engagement index and gaze entropy. Cognitive and behavioral metrics predict training outcomes with 72.5% accuracy with engagement index, gaze entropy and performance potentially all relevant to surgical skill	NASA-TLX ratings
Wu et al., 2019 [28]	Prospective observational study	n = 8	Eight surgical trainees from a large academic medical school who participated in robotic skills training	To assess the relationship between eye-tracking measures and perceived workload in robotic surgical tasks	Gaze entropy was positively correlated with the National Aeronautical and Space Administration Task Load Index (NASA-TLX) during robotic surgical tasks. Pupil diameter and gaze entropy distinguished differences in workload between task difficulty levels, and both metrics increased as task level difficulty increased. The Naïve Bayes classification Model using eye-tracking features achieved an accuracy of 84.7% in predicting workload levels	NASA-TLX ratings, Naive Bayes Model
Zia et al., 2017 [29]	Retrospective cohort study	n = 41	41 participants consisting of surgical residents and nurse practitioners	To assess a novel approach for automated assessment and analysis of Objective Structured Assessment of Technical Skills-based surgical skills	Approximate Entropy (ApEn) and Cross-Approximate Entropy (XApEn) outperform previous state-of-the-art methods using video data. For accelerometer data, the proposed method performs better for suturing only. Fusion of video and acceleration features can improve overall performance, with the proposed entropy features achieving the highest accuracy	Approximate Entropy; Cross-Approximate Entropy

**Table 2:** Lessons learned in selected articles.

Primary author/Year	Type of methodology/Analysis used
Azari et al., 2019 [12]	This study “recorded hand movements of 37 clinicians performing simple and running subcuticular suturing benchtop simulations, and applied three machine learning techniques (decision trees, random forests, and hidden Markov models) to classify surgical maneuvers every 2 s (60 frames) of video”
Brennan et al., 2019 [13]	“This prospective, non-randomized pilot study of 20 physicians at a quaternary academic medical center compared the usability and accuracy of preoperative risk-assessment between physicians and MySurgeryRisk, a validated, machine-learning algorithm, using a simulated workflow for the real-time, intelligent decision-support platform. We used area under the receiver operating characteristic curve (AUC) to compare the accuracy of physicians’ risk-assessment for six postoperative complications before and after interaction with the algorithm for 150 clinical cases”
Chapman et al., 2017 [14]	“The overall approach for this study included four steps: (1) identifying surgical patients and collecting their associated EHR data (2) annotating text data and developing a knowledge base;(3) detecting mentions of SSIs and classifying reports for the presence of a SSI and (4 evaluating automated methods against the reference standard annotations for SSIs. Institutional review board approval (IRB) was obtained”
Corey et al., 2018 [15]	“A curated data repository of postoperative outcomes was created that extracted and processed patient clinical and surgical data across 37 million clinical encounters in EHRs into 194 clinical features. Machine learning models were built off this dataset to predict risk of postoperative complications. Models were able to classify patients at high risk of postoperative complications with high sensitivity and specificity. An online calculator requiring input of 9 data fields was created to produce a risk assessment within the clinic environment”
El Chaar et al., 2019 [16]	“We created separate exploratory multivariable logistic regression models for SAEs and readmissions. We then externally validated both models using the 2016 MBSAQIP Participant Use Data File”
Hassan et al., 2015 [17]	“A total of 40 subjects without laparoscopic or robotic surgical experience were enrolled and randomized to begin with either RT or CT. Then, 2 specific RT tasks were reproduced for CT and repeated 5 times each with RT and CT. Time and quality indicators were measured quantitatively. A crossover technique was used to control for in-study experience bias”
Hernandez et al., 2014 [18]	“The Cox proportional hazards model was used to predict survival of patients with pancreatic adenocarcinoma and generated a decision model using regret-based decision curve analysis, which integrates both the patient's prognosis and the physician’s preferences expressed in terms of regret associated with a certain action. A physician's treatment preferences are indicated by a threshold probability, which is the probability of death/survival at which the physician is uncertain whether or not to perform surgery. The analysis modeled 3 possible choices: perform surgery on all patients; never perform surgery; and act according to the prediction model”
Kanitra et al., 2021 [19]	“Forty students were randomized into either Group A or B. Students practiced and were tested on a peg transfer task in either a laparoscopic simulator (LS) and robotic simulator (RS) in a pre-defined order. Performance, transference of skills and learning curve were assessed for each modality. Additionally, a fatigue questionnaire was issued”
Kulkarni et al., 2022 [20]	“Medical students performed the Fundamentals of Laparoscopic Surgery peg transfer task while recording their gaze on the monitor and tool activities inside the trainer box. Using computer vision and fixation algorithms, five scene-dependent gaze metrics and one tool speed metric were computed for 499 practice trials. Cluster analysis on the six metrics was used to group the trials into different clusters/proficiency levels, and ANOVAs were conducted to test differences between proficiency levels. A Random Forest model was trained to study metric importance at predicting proficiency levels”
Namazi et al., 2023 [21]	“The novelty of LapTool-Net is the exploitation of the correlations among the usage of different tools and, the tools and tasks-i.e., the context of the tools’ usage. Towards this goal, the pattern in the co-occurrence of the tools is utilized for designing a decision policy for the multilabel classifier based on a Recurrent Convolutional Neural Network (RCNN), which is trained in an end-to-end manner. In the post-processing step, the predictions are corrected by modeling the long-term tasks’ order with an RNN”
Parreco et al., 2018 [22]	“The Multiparameter Intelligent Monitoring in Intensive Care III database was used to obtain SICU stays with six different severity of illness scores. Natural language processing was performed on the physician notes. Classifiers for predicting mortality were created. One classifier used only the physician notes, one used only the severity of illness scores, and one used the physician notes with severity of injury scores”
Perumalla et al., 2023 [23]	“A standard deep learning architecture was used to differentiate between suture throws, knot ties, and suture cutting on a data set comprised of videos from practicing clinicians (N = 52) who participated in a simulated enterotomy repair. Perception of the added value to traditional artificial intelligence segmentation was explored by qualitatively examining the utility of identifying maneuvers in a subset of steps for an open colon resection”
Smith et al., 2022 [24]	Researchers collected 254 videos of two simulation-based exercises performed by attending surgeons. The performance in each video was scored by experienced instructors and converted into three class labels—expert, intermediate, and novice. The videos were cut into 2,227 10-second clips for training DNNs in the Google Video Intelligence AutoML service
Watson, 2014 [25]	The hand motion of the participant’s right hand was recorded while the participant was completing the latex bench model end-to-side simulated venous anastomosis. Data from the sensors to upload onto the MATLAB software environment. Each motion pattern sample was converted into a binary symbolic time series and the sequences of symbols were used as the input to calculate a Lempel–Ziv complexity score for each anastomosis. These samples were then labeled as expert or novice according to the level of training of the participant (attending surgeons were labeled expert while surgical residents were labeled novice) and used to train the SVM classification algorithm
Winkler-Schwartz et al., 2019 [26]	Through an iterative process of 5 scenarios, performance metrics associated with instrument movement and force, resection of tissues, and bleeding generated from the raw simulator (NeuroVR) data output were selected by K-nearest neighbor, naive Bayes, discriminant analysis, and support vector machine algorithms to most accurately determine group membership
Wu et al., 2021 [27]	Participants performed 12 robotic skills exercises with varying levels of difficulty repetitively in separate sessions. Electroencephalogram activity and eye movements were measured throughout to calculate three metrics: engagement index, pupil diameter and gaze entropy. Performance scores and mental workload ratings (NASA-Task Load Index) were collected after each exercise. Changes in performance scores between training sessions were calculated. Analysis of variance, repeated measures correlation, and machine learning classification were used to diagnose how cognitive and behavioral states associate with performance increases or decreases between sessions
Wu et al., 2019 [28]	Eight surgical trainees participated in 15 robotic skills simulation sessions, performing up to 12 simulated exercises. Correlation and mixed-effects analyses were conducted to explore the relationships between eye-tracking metrics and perceived workload using the NASA-TLX survey. Machine learning classifiers, like the Naïve Bayes algorithm, were used to determine the sensitivity of differentiating between low and high workload with eye-tracking features
Zia et al., 2017 [29]	The dataset of video and accelerometer data from surgical skills, knot tying and suturing, was used to evaluate the entropy-based algorithm as well as previous methods such as Sequential Motion Textures, Discrete Cosine Transform, and Discrete Fourier Transform

**Table 3:** Major challenges and outcomes of selected articles.

Primary author/Year	Lessons learned	Major outcomes	Major challenges
Azari et al., 2019 [12]	Authors believe that “the expansion and development of the methods we used could form the basis of low-cost educational tools to evaluate procedural proficiency and increase educational efficiency to ultimately improve patient safety”	“Marker-less video hand tracking can predict surgical maneuvers from a continuous video record with similar accuracy as robot-assisted surgical platforms, and may enable more efficient video review of surgical procedures for training and coaching”	“The variation in arrival rates of dynamic segments, however, would render Hidden Markov Models less appropriate for this dynamic prediction, as a movement-determined transition rate from state to state would confound the probability of transition with the underlying hand movements”
Brennan et al., 2019 [13]	“Although lacking in statistical significance for all complications, the interaction with the MySurgeryRisk algorithm resulted in a change in the physicians’ risk-perceptions and improvement in the AUC and net scores for reclassification for the tested postoperative complications. Establishing users’ attention, facilitating information processing, and updating risk-perceptions remains a challenge for all types of risk-assessment tools.” “Physician’s decision-making style can influence their perception of risk and the use of information from decision tools such as algorithms”	“Implementation of a validated, MySurgeryRisk computational algorithm for real time predictive analytics with data derived from the EHR to augment physicians’ decision making is feasible and accepted by physicians. Early involvement of physicians as key stakeholders in both design and implementation of this technology will be crucial for its future success”	The MySurgeryRisk Algorithm used data only taken from a single medical center with a small sample physician sample size (n = 20). Furthermore, the physician risk-assessment can be positively influenced by the MySrugeryRisk algorithm in terms of finding predictive information within the patient’s electronic health record
Chapman et al., 2017 [14]	“When utilizing linguistic and semantic context, fcClassifier maintained a high recall (0.93) while achieving a much higher precision (0.82). This high precision could enable a more accurate selection of reports for review, greatly reducing the amount of work needed to evaluate and detect surgical site infections. This demonstrates the value of including contextual features when extracting information from clinical reports”	“The automated detection of adverse surgical event has the potential to revolutionize how providers and hospitals detect and report quality measures. Currently, these efforts rely on manual chart review which limit the scalability and generalizability of quality measurement activities. In addition, leveraging automated detection into clinical decision support services may lead to better surveillance and the potential to improve patient care. We executed automated detection of evidence of intraabdominal surgical site infections using a rule-based NLP system that achieved accuracy similar to manual chart review. In addition, this system out-performed other approaches using administrative data or SVM machine learning techniques”	The surgical patients evaluated in the study were taken from reports of the MIMICIII Critical Care Database, which implies that a large majority of the patients were in critical settings. This can result in a larger number of surgical site infections in comparison to the general populations identified by the software. Furthermore, the scope of the project was limited as the study only analyzed CT radiology reports of one anatomical region with one target phrase (“fluid collection”)
Corey et al., 2018 [15]	Machine learning models built off an automatically extracted and curated EHR surgical dataset have strong predictive performance for detecting surgical complications. The long-term cost of updating this surgical dataset is lower than that of manually updated datasets	“Extracting and curating a large, local institution’s EHR data for machine learning purposes resulted in models with strong predictive performance. These models can be used in clinical settings as decision support tools for identification of high-risk patients as well as patient evaluation and care management. Further work is necessary to evaluate the impact of the Pythia risk calculator within the clinical workflow on postoperative outcomes and to optimize this data flow for future machine learning efforts”	The project had a larger number of missing data from encounters resulting in a significant loss of the sample size of the invasive procedures. Furthermore, the complications identified within the models were also broadly defined, limiting the user’s ability to understand exactly which type of complication the patient is at risk for within the groupings
El Chaar et al., 2019 [16]	Significant predictors of SAEs were preoperative body mass index, GB surgery, cardiovascular disease, smoking, diabetes, hypertension, limited ambulation, sleep apnea, history of pulmonary embolism, and steroid use. Significant predictors of readmissions were GB surgery, female sex, diabetes, hypertension, preoperative body mass index, sleep apnea, history of pulmonary embolism, cardiovascular disease, smoking, and limited ambulation. External validation supported these covariates, with similar model discriminative power”	“Using the MBSAQIP database in a novel way, we developed clinical risk prediction models for SAEs and readmissions in the first 30 days following bariatric surgery. Although these risk models may prove valuable for the purposes of patient counseling, risk stratification, and preoperative medical optimization, they should be used and interpreted taking into account local surgeon expertise and experience, as well as other variables that may affect patients’ surgical risks and subsequent outcomes”	The data included in the manuscript did not include any significant complications outside of the intervention or reoperation. The models also did not record any surgical complications that occurred 30 days after the procedure. Furthermore, the data comes with the drawbacks of a retrospective cohort study while also not being able to generalize to patients outside of North America
Hassan et al., 2015 [17]	“We found that speeds were significantly faster overall with RT than with CT, but that the percentage of speed improvement with increasing trials was similar for both RT and CT. We did not find that prior experience with one modality translated into a more efficient usage of the other. Both RT and CT play an important role in augmenting operative skills in minimally invasive surgery, with CT being comparable to RT in efficiency, as a training platform, and even more applicable as a training tool, especially for general surgical residents such as in our study”	“Since its superior handling, better vision, and other ergonomic benefits lead to consistently faster completion of tasks, RS is more intuitive than CLS, though the lack of tactile feedback in the robotic manipulators may cause suboptimal outcomes, especially in naive users”	A more clinical comparison of learning curves between robotic and standard laparoscopic instrumentations could have been made. A quantitative measurement of technical skill is hard to analyze in the context of the software. Measurement and collection of real-time motion data for corresponding laparoscopic tasks was still a technical challenge in the study
Hernandez et al., 2014 [18]	“Significant independent predictors of overall survival included preoperative stage and pathological stage. Compared with the ‘always aggressive’ or ‘always passive’ surgical treatment strategies, the survival model was associated with the least amount of regret for a wide range of threshold probabilities”	“Significant independent predictors of overall survival included preoperative stage and pathological stage. Compared with the ‘always aggressive’ or ‘always passive’ surgical treatment strategies, the survival model was associated with the least amount of regret for a wide range of threshold probabilities”	“Regret theory” was applied retrospectively with assigned cutoff survival values, assuming that maximal regret operating on a patient would be in one who died within the first 7 months after resection. This approach has not yet been empirically tested and the prediction model has not been externally validated. In designing this model it was assumed that there is a single decision maker involved in the process where, in actual practice, a multidisciplinary team of health care providers is involved in treatment decisions
Kanitra et al., 2021 [19]	“There was no significant difference between overall laparoscopic scores and robotic scores Prior laparoscopic skills performed significantly better on robotic testing than without laparoscopic skills There was no significant difference in scores between students with prior robotic skills than without robotic skills Students reported no difference in fatigue between RS and LS The learning curve plateaus at similar times between both modalities”	“Novice medical students with laparoscopic skills performed better on a RS test than students without laparoscopic training, suggesting a transference of skills from laparoscopic to robotic surgery. These results suggest laparoscopic training may be sufficient in general surgery residencies as the skills transfer to robotic if used post-residency”	This study had relatively small power. The use of novice medical students may have been the reason for significant results due to less surgical experience and greater ability to improve in a shorter period of time. The single institution design may affect the generalizability of the results. Only one task was performed, which is not representative of a comprehensive skills assessment
Kulkarni et al., 2022 [20]	“Our results are consistent with the literature in that trials at most proficient level exhibited the best gaze metrics, slowest tool speed, and fastest completion time. Advances in utilizing scene-dependent gaze metrics are particularly important for formative assessment at informing trainees where to look (or where they have not been looking) and thereby shaping their subsequent practice strategies or behaviors This research presents two sensitive feedforward eye gaze metrics which have revealed that trainees in their highest proficiency trials looked ahead more often and looked less at objects and tools under their manipulation”	“Scene-dependent gaze metrics could reveal skill levels more precisely than between experts and novices as suggested in the literature. Taken together, this research provides further insight into changes in gaze behaviors with respect to proficiency levels in laparoscopic surgical skills and is applicable to the development of an automated tool that would accelerate trainee skill acquisition”	This study assumes that completion time as a performance criterion in defining skill level which may be necessary but insufficient. The study only recruited medical students given the research focus on skill acquisition, further verification is necessary to assess the degree to which these scene-dependent gaze metrics reflect expertise of surgeons who recently perform many or few minimal invasive procedures.
Namazi et al., 2022 [21]	“The relatively small training set after under-sampling suggests that the labeling process can be accomplished faster by using fewer frames The simple architecture of the proposed LP-based classifier makes it easy to use it with other proposed models. The tool combinations might slightly vary for each operation, which suggests that the combination patterns can be surgeon/ patient dependent; therefore, the LP-based decision policy might not be suitable if detecting the rare combinations is of priority”	“The results show LapTool-Net outperformed state-of-the-art methods significantly, even while using fewer training samples and a shallower architecture. Our context-aware model does not require expert’s domain-specific knowledge and the simple architecture can potentially improve all existing methods. However, further development would be required for detection of rare or nuanced combinations”	This tool requires all the possible combinations of tools to be present in the training set to make the correct prediction on all of them, making detection of procedures with more tool variability more difficult
Parreco et al., 2018 [22]	“The classifiers were able to detect the subtle nuances in physician vernacular that predict mortality. These nuances provided improved performance in predicting mortality over physio-logic parameters alone”	“This study demonstrates the novel use of artificial intelligence to process physician documentation to predict mortality in the SICU”	Spelling mistakes and utilization of acronyms in surgeon notes created variation in the usability of this model
Perumalla et al., 2023 [23]	“The precision for knot ties was 60%. We suspect that the precision was low due to the lack of data for this class compared with the suture throws and suture cuts, where there was more data (hence the higher precision). In the future, training the model with more annotated knot tying videos could improve the precision. The trained model that has been described and presented in this work may not be useful in identifying error management and safety measures in certain other use cases, but the video collection protocol, annotation, and model architecture that was chosen in our work can be leveraged in these cases”	“AI-enabled video segmentation tools aim to facilitate quick and focused reviews by segmenting the videos according to standard procedural steps. However, real-life surgeries are complex. Procedural steps, which are based on ideal protocols, may not shed light on case complexity and decision-making. We have demonstrated that a reasonably accurate AI-enabled method to automatically detect basic maneuvers can be developed and tested on a data set of simulated enterotomy repair videos. A qualitative concept mapping was demonstrated to show that real-life scenarios can benefit from the identification of basic maneuvers”	The trained model that has been described and presented in this work may not be useful in identifying error management and safety measures in certain other use cases as error management varies per procedure
Smith et al., 2022 [24]	“The algorithms could be much more accurate with more video clips to learn from. We chose to cut the exercise videos into 10s clips. These original videos could be cut into 5 s or 3 s clips to create a dataset that is two to three times larger. However, it is important that these shorter clips present an action that truly demonstrates differentiable levels of skill. If these shorter videos provide more instances of activities that look the same at every skill level, then they would not improve the performance of the algorithm”	The GEARS overall score with an average accuracy of 83.1% with a balance of precision (Type I error) of 76.1% and recall (Type II error) of 67.7% (Table 3). The DNN model for the Suture Sponge exercise achieved an average accuracy of 80.8%, precision of 72.8%, and recall of 67.7%	Experienced AI scientists prefer to address the misclassification problem by adjusting the thresholds or by providing larger datasets for training. For this project, we classified the videos using the standard surgical skills levels of expert, intermediate, and novice. However, if the model is only being used to determine whether a student has improved beyond a defined skill level to pass an educational course, the classifications could be simplified to a binary pair such as Pass/Fail
Watson, 2014 [25]	Authors believe that the expansion and development of the methods we used could form the basis of low-cost educational tools to evaluate procedural proficiency and increase educational efficiency to ultimately improve patient safety	Support vector machine (SVM) algorithm increased the predictive power to classify blinded surgical hand motion patterns into expert versus novice groups. The SVM algorithm had an accuracy of 83% (sensitivity 86%, specificity 80%)	This study does have significant limitations. The study sample size was small and from a single institution, and the participating faculty and residents were volunteers rather than a random sample. We did not examine the quality of the finished anastomoses and have not proved that the classifier is an indicator of the quality of task outcome. We did not evaluate real surgical skill, and we assumed that attending surgeons had more surgical skill and residents had less
Winkler-Schwartz et al., 2019 [26]	Simulation-based technical skills training informed by artificial intelligence feedback systems may offer an alternative to human evaluators	The machine learning algorithm successfully classified participants into four levels of expertise with 90% accuracy with as few as six performance metrics	Although four different machine learning algorithms were used, there still exists the possibility that all algorithms are overfitted to our data set, limiting their performance when faced with novel data. In response to this misclassification, we sought to limit misclassifications between these two groups in the algorithm optimization process as a proof of concept. In addition, it is challenging to define populations of surgeons, fellows, and residents with equivalent skill to allow accurate classification
Wu et al., 2021 [27]	Factors that influence performance changes can be multifaceted but sensor-based metrics can complement current feedback tools for predicting training performance. This demonstration allows the possibility of using real-time measurement to monitor the training process and providing assessments other than task performance score	Changes in performance were negatively correlated with changes in engagement index and gaze entropy. Cognitive and behavioral metrics predict training outcomes with 72.5% accuracy with engagement index, gaze entropy and performance potentially all relevant to surgical skill	Task order was not randomized and trainee schedules and time between sessions were not controlled. Moreover, the small number of participants could impact generalizability
Wu et al., 2019 [28]	Eye-tracking metrics can identify difficult phases during training and help with the curriculum design and identify trainees who are experiencing unusually high workload and are in need of extra help	Gaze entropy was positively correlated with National Aeronautical and Space Administration Task Load Index (NASA-TLX) during robotic surgical tasks. Pupil diameter and gaze entropy distinguished differences in workload between task difficulty levels, and both metrics increased as task level difficulty increased. The Naïve Bayes classification Model using eye-tracking features achieved an accuracy of 84.7% in predicting workload levels.	Task orders were not randomized and the number of sessions and exercises for each participant were not controlled
Zia et al., 2017 [29]	Videos are better for extracting skill relevant information as compared to accelerometer. However, a fusion of video and accelerometer features can improve performance	Approximate Entropy (ApEn) and Cross-Approximate Entropy (XApEn) out-perform previous state-of-the-art methods using video data. For accelerometer data, the proposed method performs better for suturing only. Fusion of video and acceleration features can improve overall performance with the proposed entropy features achieving highest accuracy	ApEn and XApEn work better for high dimensionality data, which can lead to potential over-fitting, and can be computationally expensive

Steps 4 and 5. Data Charting and Collation, Summarization, and Reporting of Results

Table 1 was tabulated to present study characteristics, including primary author, country where the study was conducted, study design, sample size, study population, age range of participants, study purpose, and their major findings. Study-specific results were identified and highlighted in Table 2. For Table 3, to analyze themes in future recommendations identified in the primary qualitative research, the three phases of qualitative content analysis described by Elo and Kyngas were applied: (i) preparation, (ii) organizing, and (iii) reporting. For the preparation phase, a theme was first chosen as a unit of analysis [30]. This was followed by the organization phase of sorting qualitative data using open coding, in which themes were documented while reading through the papers, the creation of general categories from extracted themes, and the abstraction to organize generic categories into higher-order classes. A categorization matrix was developed to code the data according to the categories and perform deductive content analysis. The final step was reporting the analysis process and results in Table 3.

Results

Study Selection and Characteristics

**Figure 1:**
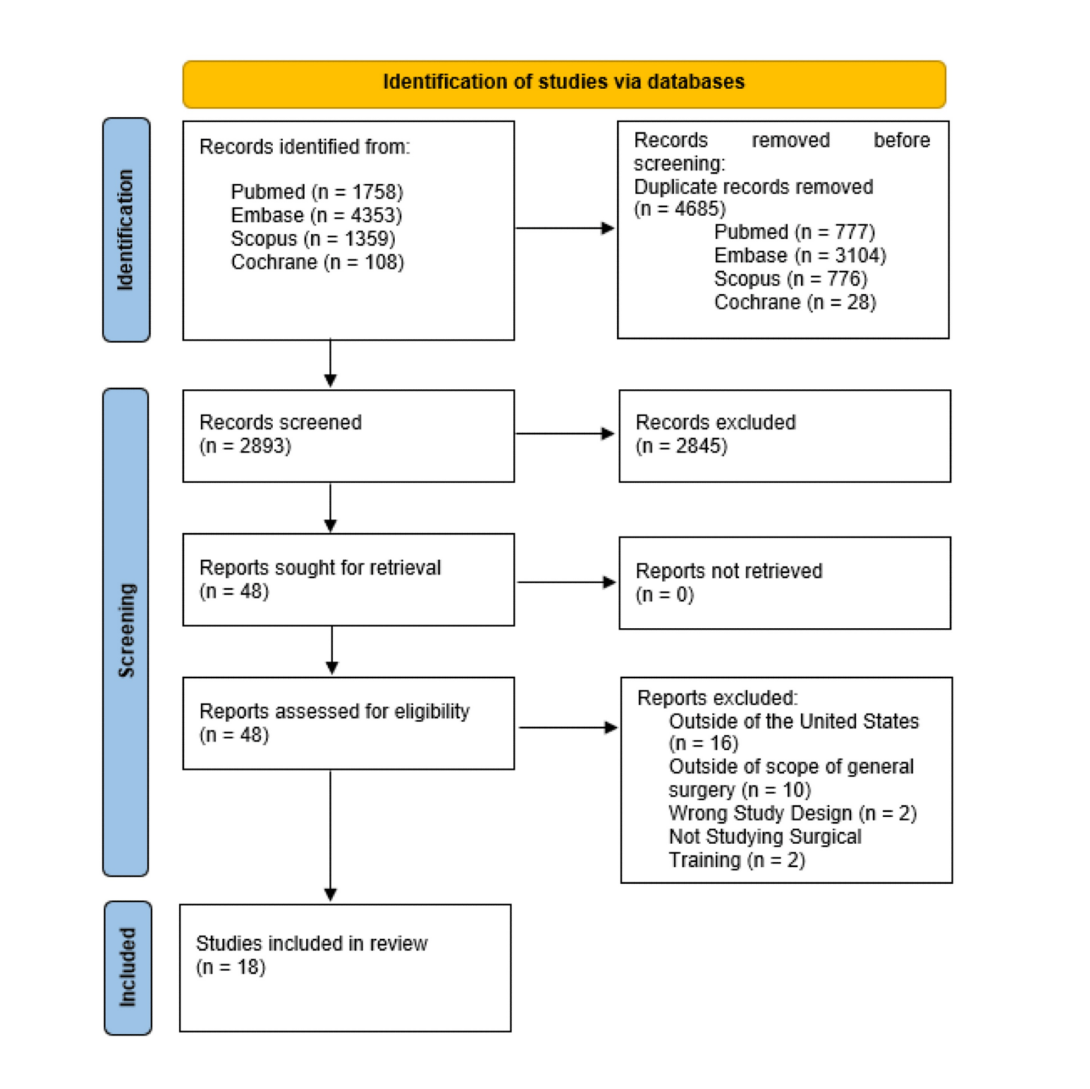
Preferred Reporting Items for Systematic Reviews and Meta-Analyses (PRISMA) flow diagram. The PRISMA flow diagram for the scoping review detailing the database searches, the number of articles screened, and the full texts retrieved and included in the study.

As seen in Figure 1, the initial study extraction resulted in 7,578 articles from PubMed (n = 1,758), EMBASE (n = 4,353), Cochrane (n = 108), and Scopus (n = 1359). Studies were excluded due to utilizing the wrong study design, including published abstracts, narrative reviews, scoping reviews, systematic reviews, and editorials, not focusing on general surgery training, such as articles examining plastic surgery, urology, neurosurgery, or orthopedic surgery training, being performed outside of the U.S, being performed before 2014, and being performed on patients under the age of 18 (n = 2,875). Duplicate studies were also excluded (n = 4,685). A total of 18 met the inclusion criteria from PubMed (n = 5), EMBASE (n = 5), Cochrane (n = 2), and Scopus (n = 6). The 18 included studies were published between 2014 and 2024. Study designs included prospective cohort studies (n = 6), qualitative studies (n = 5), retrospective cohort studies (n = 4), randomized controlled trials (n = 2), and an observational study (n = 1). Sample sizes ranged from n = 7 to n = 101,599.

Synthesis of Results

The reviewed studies demonstrated AI’s utility in two primary areas, namely, clinical decision support and technical skill assessment. For example, Brennan et al. (2019) used AI to test a data-driven algorithm (MySurgeryRisk) for predicting post-operative protocols and standards of care. With the relative ease of using such a protocol, the physicians of the study were able to increase the identification of post-operative kidney disease and intensive care unit admissions by 12% and 16%, respectively, in comparison to initial risk assessment [13]. Another method of AI usage came through a teaching and monitoring mechanism implemented by Zia et al. (2017) (Objective Structured Assessment of Technical skills), which helped better train personnel on different surgical procedures by lowering different entropic actions and maximizing the efficiency of procedures for these students. With the program analyzing the video of the trainees, it was successfully able to classify 95.1% of suturing actions and 92.2% knot-tying actions [29]. Similar AI training programs were used in studies by Winkler-Schwartz et al. (2019), whose algorithm was able to differentiate and classify participants according to their level of practice in surgery with an accuracy of 90% [26].

Other studies used AI to observe various physical variables (i.e., eye movement, behavioral metrics, and linguistic standards) to correlate them with surgical performance, training, and deficits [14,28]. For example, Wu et al. (2019) observed the eye movement of the surgical trainees as a measure of increasing stress levels for the students during surgery. The eye-tracking features were able to achieve 84.7% accuracy in terms of predicting workload levels, with increased intensities of workload and stress [28]. Chapman et al. (2017) analyzed physician notes to study how different linguistic trends can be used to predict the quality of care patients are receiving pre- and postoperatively. This protocol was able to accurately identify fluid collection in charting record analysis with 0.92 recall and 0.90 precision [14]. The surgical sector of medicine has shown how many diverse ways AI can be implemented into the field, by helping make progress in the training, teaching, and treatment aspects of patient care. Overall, most of the articles reviewed (n = 12) focused on the implementation of AI to help with procedural skills specifically. Other articles focused on implementing AI for aiding in patient care before and after surgery (n = 4) and analyzing surgical notes (n = 2).

Each of the articles reviewed in our study showed the different potential that AI has in the surgical field, highlighting the multiple facets of this sector that require improvement or configuration. For example, in the study done by Azari et al. (2019), video hand tracking proved to show beneficial results compared to robot-assisted surgeries in terms of procedural review through video tracking software that classified different surgical maneuvers in real-time. By recording the accuracy of these video analysis results, this helps better train students in the surgical field by reviewing these videos of the procedures and examining specific needs for improvement. The AI software classified different actions of the procedure into categorical variables that allowed for specific markers to be reviewed by the student postoperatively. With AI implemented into video observation during these procedures, selected components can be monitored and studied further to improve training standards for students [12].

In the study reported by Corey et al. (2018), the designed ML models analyzed and updated electronic health record (EHR) data sets to better predict complications of postoperative patients [15]. These models more efficiently and effectively altered the pre-existing datasets of EHR compared to manual updates, allowing for quicker updates to any standards of care. With AI possibly being integrated into EHR datasets, physicians of all specialties, not only surgery, can have diagnosis and treatment protocols updated promptly. Along with similar guidelines, another study by Hernandez et al. (2014) showed how different predictors of survival were formulated into a model for the preoperative and pathological stage of patient care. These models showed how implementing different treatment strategies can be correlated with different thresholds of probability for survival. Once again, these predictive models can be focused on improving different treatment methods to ensure that the survival probability of patients is maximized, which makes AI a beneficial tool for using short-term data collection for constant analysis [18]. With such a plethora of modes of possible AI integration into the surgical field demonstrated by these articles, the potential of AI in healthcare cannot be underestimated.

There were also significant limitations that were shared between the articles, according to Table 3. First, a large majority of the articles within our project’s purview did not have a large enough sample size to generalize the findings or efficiency of the ML/AI used within the study. This could have been because of the lack of participants in the study, the number of possible data points to be analyzed, or the specificity of the data that needed to be analyzed for the study. Another common complication, specifically for articles regarding technical procedural skills, was difficulty in coordinating the AI with the real-time motion of the surgeon performing the procedure. This made it harder for the data to be properly collected in a manner that could be analyzed appropriately.

Certain studies in our review compiled data from underqualified individuals practicing or earning surgical procedures (i.e., medical students), which could not be properly assessed for their work in the study. This speaks to the notion that such machine learning and AI frameworks would need to be standardized with professional expertise during procedures. Lastly, several studies in our review mentioned concerns about not being able to review a wide variety of the procedural skills needed to be assessed. Instead, the limited framework of the data analysis could only be used to review the data regarding one skill, procedure, or dependent variable. This could be due to the limited scope of data analysis of the AI used within each study.

Discussion

This scoping review provided greater insight into the use of AI and ML in general surgery training by exploring the use of these tools in this field in the United States within the last 10 years. Through the analysis of 18 studies, this review investigated the wide range of utility in the application of ML and AI, including but not limited to proficiency evaluation, risk determination, and procedural assessment. The findings provide a comprehensive overview and increased awareness of the state of AI and ML applications in general surgery training today. These findings provide valuable insights and recommendations for future applications of these tools not only in this surgical field, but throughout the other medical specialties as well. One of the aims of this review was to gather the current trends, challenges, and opportunities in the integration of AI and ML technologies within surgical training. A common goal among the papers selected was to evaluate the ability of AI to aid in the assessment of surgical skills and complex maneuvers in an automated, standardized manner.

One of the most consistently reported trends was the use of AI to identify specific surgical maneuvers in real-time. Studies such as those by Azari et al., as well as Perumalla et al., utilized video analysis and hand-tracking technologies to classify surgical actions, such as knot tying and suturing [12,23]. Similarly, studies by Zia et al. implemented video-based classification of procedural tasks aimed at reducing subjectivity in surgical education [29]. Across the board, ML models were able to successfully track simple maneuvers in real-time with accuracy. However, increased difficulty was encountered in tracking more advanced maneuvers, especially in the transition between one maneuver to another [12]. The studies suggest that as these models advance, real-time detection systems could eventually be integrated into curriculum development to create unbiased feedback and targeted training suggestions for surgical trainees [21]. This unbiased and standardized feedback, along with expert surgeon feedback, provides trainees with enough information to address their deficiencies in performance.

Another major trend among these studies related to the classification of surgical proficiency using ML models. Studies by Smith et al., Watson et al., and more utilized support vector machines and deep learning models to demonstrate the feasibility of distinguishing between different skill levels in the operating room with accuracy [24,25]. Winkler-Schwartz et al. extended this approach by developing an ML model capable of classifying trainees into four levels of surgical expertise using only six performance metrics in a virtual reality environment [26]. These findings support the use of AI and ML in developing data-driven assessment tools that can supplement human evaluation. The potential synergy between holistic human evaluation and standardized, data-driven machine feedback offers a promising path toward integrating algorithmic precision with human intuition to enhance surgical training and patient care [25].

Common challenges in creating and using AI and ML models for surgical training were seen across the various studies included in this review. A key challenge seen in the design of multiple ML models was overfitting to the provided data set [12,20]. Overfitting is defined as the phenomenon where a highly predictive model on the training data generalizes poorly to other data sets or future observations [27]. A number of studies found this to be the case at the level of detecting simple tasks up to classifying the steps of a complex procedure [12]. Furthermore, considering the frequently encountered variability between surgeons performing the same type of surgery, this outcome ultimately limits the generalizability of these ML models for widespread use. This challenge remains one of the key hurdles in the adoption of AI, not only from the perspective of the surgical training concept, but across many other industries as well.

Another concept this review aimed to expand on was the future applications of AI and ML technologies in surgical training. Many of the included studies agree that the combination of identifying surgical maneuvers in real time with the classification of surgical proficiency would not only aid in skill tracking and curriculum development but also facilitate targeted training, allowing advanced surgeons to pinpoint where a learner may improve during the operative process. For example, if an AI system detects repeated struggles or inconsistencies when suturing, it can provide targeted information or practice simulations focused on improving that specific skill. They also suggest possible utility for advanced surgeons as well, through the integration of AI in robotic surgery platforms to alert surgeons to deviations from expected patterns, or recommend optimal maneuvers based on accumulated data [12]. This intraoperative decision support may ultimately enhance safety and potentially improve patient outcomes.

Another avenue in which AI may be integrated into surgical training is in the analysis of non-technical performance metrics that impact operative capability. For example, Wu et. al used eye-tracking data to assess cognitive load during surgery, achieving high accuracy in predicting workload levels. This could help educators understand how stress affects performance and offer targeted interventions to improve trainee resilience and cognitive focus. Though these models remain in development, future application in clinical environments will provide even greater information and insight into the usage and challenges of these programs [28].

The final goal of this study was to determine the impact of AI and ML on the diagnostic acumen and clinical decision-making abilities of surgical trainees. We found that a number of studies focused on the use of AI in determining pre- and post-operative risk predictions, an important competency for a surgical trainee, especially in their early years of training. AI-driven tools such as MySurgeryRisk have demonstrated tangible benefits in patient care by improving the accuracy of postoperative risk predictions. The algorithm enabled clinicians to better anticipate post-operative complications compared to traditional risk assessments. Though not integrated into surgical training curricula yet, these predictive models may help guide postoperative protocols and standards of care as they are integrated into health systems that will ensure more timely and appropriate interventions that directly benefit patient outcomes [13]. Chapman et al. used natural language processing to analyze physician notes and identify clinical trends. Their algorithm successfully predicted fluid collections with great precision, demonstrating how AI can support improved diagnostic accuracy through better documentation analysis [14]. Integration of these models in the United States is still in its early stages, as these models continue to be developed and refined; however, their future implementation has a strong likelihood of aiding trainees in clinical decision-making and developing diagnostic acumen.

Though this study focused on general surgery training, AI and ML applications are not limited to training in this specific field. Throughout this review, we encountered many studies looking at the use of these tools in other medical specialties. For example, in the field of oncology, Feng and colleagues developed and evaluated a deep learning network for parotid gland tumor diagnosis via deep learning MR images. The model was able to accurately distinguish between benign and malignant tumors, suggesting usefulness when used in conjunction with clinical reasoning [31]. Another example is seen in a 2018 urological study by Jelovsek et al., where the researchers constructed and validated a prediction model for estimating the risk of de novo stress urinary incontinence after vaginal pelvic organ prolapse surgery. They compared their model’s predictions with established prediction methods, such as preoperative urinary stress testing and expert surgeons’ predictions, ultimately finding that their model outperformed all the established methods of predicting this complication after surgery [32]. Overall, similar themes such as AI being efficacious in risk assessment, surgical skill proficiency evaluation, and procedural detection are found throughout this vast pool of studies as well. Further studies should examine the utilization of AI in other surgical specialties as well as nonsurgical medical specialties.

A number of limitations were encountered during the completion of this study. The limitations of this study can be attributed to a number of factors, including the study design and variability of article methods and results. First, the review focused on only analyzing studies specifically from the United States surgical training system. Even though this was an intentional measure, it excludes relevant and high-quality research regarding AI in surgery from other countries around the world. Although the screening process implemented in this study was particular and detailed, it was challenging to standardize a single method in which AI was implemented in the surgical field. With such a wide variety of ways that AI can be used in the medical field, each of the studies we analyzed focused on different independent and dependent variables. This makes quantitative analysis of these studies more cumbersome. Although generalized themes of AI competency can be investigated, finding specific numerical changes in AI-related interventions is difficult to standardize within our studies. Furthermore, the scoping parameters used within this were challenging to establish due to concerns about our scope being too broad or too narrow. Defining what surgeries can be considered in the realm of “general surgery” was difficult to establish after the search terms were inputted. Although another thorough review was done to ensure no other relevant articles were lost, ensuring that all possible articles involved in the scope of our research were present was challenging.

This research provided valuable insights into the state of AI and ML use in American General Surgery training; however, limiting this research to training programs within the United States narrowed the scope of this study, allowing for the inclusion of 18 studies that fit the criteria. AI implementation in surgical training is a booming topic of study globally; therefore, future studies assessing the advancements of AI and ML implementation within this niche would provide a more comprehensive and inclusive overview of the innovative ways these tools are being utilized. For example, a study by Mansour and colleagues compared six established convolutional neural network models in objectively assessing medical trainee suturing skills, which were found to be extremely accurate in this task [33]. Including the innovative approaches of studies from other nations would provide an even more comprehensive and encompassing outlook on AI and ML advances. Areas of focus for future research include creating new ML models for tasks at various points in surgery, strengthening established AI or ML models for detection and assessment of procedure steps and proficiency, and applying AI assessment tools in learning spaces and assessing their success in teaching outcomes.

## Conclusions

With the rapid pace at which AI is progressing around the world, the medical field has plenty to gain from implementing AI into the way that patients are treated. Specifically, the surgical sector of healthcare, with its rigorous training and complex procedures, can benefit from the aid of AI being integrated through all facets of healthcare. This review reaffirms the wide variety of ways that AI can be adapted to help with facets of surgery, including training residents and students, providing optimal pre- and post-surgical care, and fine-tuning EHR systems to make physician-patient interactions more effective. Although AI still has many strides to be applied throughout all aspects of surgery, we believe that it certainly has the potential to be used in specific settings and situations.

## Appendices

The search string we input for each of the databases we reviewed for our research is available below:

Database: PubMed.gov (Includes MEDLINE) 2,999 (2014-2024)

(“INSERT MESH TERM”[Mesh] OR “INSERT MESH TERM”[Mesh] OR (“INSERT SYNONYM”[tiab] OR “INSERT SYNONYM”[tiab] OR “INSERT SYNONYM”[tiab] OR “INSERT SYNONYM”[tiab] OR “INSERT SYNONYM”[tiab] OR “INSERT SYNONYM”[tiab] OR “INSERT SYNONYM”[tiab] OR “INSERT SYNONYM”[tiab]))

AND

(“INSERT MESH TERM”[Mesh] OR “INSERT MESH TERM”[Mesh] OR (“INSERT SYNONYM”[tiab] OR “INSERT SYNONYM”[tiab] OR “INSERT SYNONYM”[tiab] OR “INSERT SYNONYM”[tiab] OR “INSERT SYNONYM”[tiab] OR “INSERT SYNONYM”[tiab] OR “INSERT SYNONYM”[tiab] OR “INSERT SYNONYM”[tiab]))

(“Artificial Intelligence”[Mesh] OR “Machine Learning”[Mesh] OR “Deep Learning”[Mesh] OR “Supervised Machine Learning”[Mesh] OR “Unsupervised Machine Learning”[Mesh] OR (“Artificial Intelligence”[tiab] OR “AI”[tiab] OR “Machine Learning”[tiab] OR “Deep Learning”[tiab] OR “automated identification”[tiab] OR “predictive model”[tiab] OR “prediction model”[tiab] OR “ensemble models”[tiab] OR “ensemble learning”[tiab] OR “ensemble methods”[tiab] OR “risk prediction”[tiab]))

AND

(“General Surgery”[Mesh] OR “Surgical Procedures, Operative”[Mesh] OR “Specialties, Surgical” [Mesh] OR “Early Detection of Cancer”[Mesh] OR “Neoplasm Metastasis”[Mesh] OR (“Surgery”[tiab] OR “Surgical”[tiab] OR “Cancer screening*”[tiab] OR “Cancer diagnos*”[tiab] OR “Malignant”[tiab] OR “Metastasis”[tiab]))

AND

(“Education, Medical, Graduate” [Mesh] OR “Internship and Residency”[Mesh] OR (“Clinician*”[tiab] OR “Physician*”[tiab] OR “Graduate Medical Education”[tiab] OR “surgery resident*”[tiab] OR “surgery residency*”[tiab] OR “surgical residency*”[tiab] OR “surgical resident*”[tiab] OR “Surgical Trainee*”[tiab] OR “Surgery Trainee*”[tiab] OR “Surgical Training*”[tiab] OR “Surgery Training*”[tiab] OR “Medical Training*”[tiab] OR “Residency Training*”[tiab] OR “surgical education”[tiab] OR “surgery education”[tiab]))

AND

(“Data Accuracy”[Mesh] OR “Prognosis”[Mesh] OR “Diagnosis”[Mesh] OR “Early Diagnosis”[Mesh] OR “Risk Assessment”[Mesh] OR (“survival outcomes”[tiab] OR “accuracy”[tiab] OR “Prognosis”[tiab] OR “Prognostic Accuracy”[tiab] OR “Diagnosing”[tiab] OR “Diagnosis”[tiab] OR “diagnostic accuracy”[tiab] OR “Risk Assessment”[tiab]))

Database: Embase.com (Elsevier) 5,876 (2014-2024)

(‘Artificial Intelligence’/exp OR ‘Machine Learning’/exp OR ‘Deep Learning’/exp OR ‘predictive model’/exp OR (‘Artificial Intelligence’:ti,ab OR AI:ti,ab OR ‘Machine Learning’:ti,ab OR ‘Deep Learning’:ti,ab OR ‘automated identification’:ti,ab OR ‘predictive model’:ti,ab OR ‘prediction model’:ti,ab OR ‘ensemble models’:ti,ab OR ‘ensemble learning’:ti,ab OR ‘ensemble methods’:ti,ab OR ‘risk prediction’:ti,ab)) AND (‘Surgery’/exp OR ‘cancer screening’/exp OR ‘cancer disagnosis’/exp OR ‘Metastasis’/exp OR (Surgery:ti,ab OR Surgical:ti,ab OR ‘Cancer screening*’:ti,ab OR ‘Cancer diagnos*’:ti,ab OR Malignant:ti,ab OR Metastasis:ti,ab)) AND (‘Medical education’/exp OR ‘Resident’/exp OR ‘surgical training’/exp OR ‘Residency training’/exp OR (Clinician*:ti,ab OR Physician*:ti,ab OR ‘Graduate Medical Education’:ti,ab OR ‘surgery resident*’:ti,ab OR ‘surgery residency*’:ti,ab OR ‘surgical residency*’:ti,ab OR ‘surgical resident*’:ti,ab OR ‘Surgical Trainee*’:ti,ab OR ‘Surgery Trainee*’:ti,ab OR ‘Surgical Training*’:ti,ab OR ‘Surgery Training*’:ti,ab OR ‘Medical Training*’:ti,ab OR ‘Residency Training*’:ti,ab OR ‘surgical education’:ti,ab OR ‘surgery education’:ti,ab)) AND

(‘Accuracy’/exp OR Prognosis/exp OR Diagnosis/exp OR ‘Diagnosis accuracy’/exp OR ‘Risk Assessment’/exp OR (‘survival outcomes’:ti,ab OR accuracy:ti,ab OR Prognosis:ti,ab OR ‘Prognostic Accuracy’:ti,ab OR Diagnosing:ti,ab OR Diagnosis:ti,ab OR ‘diagnostic accuracy’:ti,ab OR ‘Risk Assessment’:ti,ab))

AND

(2014:py OR 2015:py OR 2016:py OR 2017:py OR 2018:py OR 2019:py OR 2020:py OR 2021:py OR 2022:py OR 2023:py OR 2024:py)

Database: Cochrane Library 178 trials, 4 Reviews (2014-2024)

([mh “Artificial Intelligence”] OR [mh “Machine Learning”] OR [mh “Deep Learning”] OR [mh “Supervised Machine Learning”] OR [mh “Unsupervised Machine Learning”] OR (“Artificial Intelligence”:ti,ab OR AI:ti,ab OR “Machine Learning”:ti,ab OR “Deep Learning”:ti,ab OR “automated identification”:ti,ab OR “predictive model”:ti,ab OR “prediction model”:ti,ab OR “ensemble models”:ti,ab OR “ensemble learning”:ti,ab OR “ensemble methods”:ti,ab OR “risk prediction”:ti,ab)) AND ([mh “General Surgery”] OR [mh “Surgical Procedures, Operative”] OR [mh “Specialties, Surgical”] OR [mh “Early Detection of Cancer”] OR [mh “Neoplasm Metastasis”] OR (Surgery:ti,ab OR Surgical:ti,ab OR (“Cancer” NEXT screening*):ti,ab OR (“Cancer” NEXT diagnos*):ti,ab OR Malignant:ti,ab OR Metastasis:ti,ab)) AND ([mh “Education, Medical, Graduate”] OR [mh “Internship and Residency”] OR (Clinician*:ti,ab OR Physician*:ti,ab OR “Graduate Medical Education”:ti,ab OR (“surgery” NEXT resident*):ti,ab OR (“surgery” NEXT residency*):ti,ab OR (“surgical” NEXT residency*):ti,ab OR (“surgical” NEXT resident*):ti,ab OR (“Surgical” NEXT Trainee*):ti,ab OR (“Surgery” NEXT Trainee*):ti,ab OR (“Surgical” NEXT Training*):ti,ab OR (“Surgery” NEXT Training*):ti,ab OR (“Medical” NEXT Training*):ti,ab OR (“Residency” NEXT Training*):ti,ab OR “surgical education”:ti,ab OR “surgery education”:ti,ab)) AND ([mh “Data Accuracy”] OR [mh Prognosis] OR [mh Diagnosis] OR [mh “Early Diagnosis”] OR [mh “Risk Assessment”] OR (“survival outcomes”:ti,ab OR accuracy:ti,ab OR Prognosis:ti,ab OR “Prognostic Accuracy”:ti,ab OR Diagnosing:ti,ab OR Diagnosis:ti,ab OR “diagnostic accuracy”:ti,ab OR “Risk Assessment”:ti,ab))

Scopus 2,844 (2014-2024)

(INDEXTERMS(“Artificial Intelligence”) OR INDEXTERMS(“Machine Learning”) OR INDEXTERMS(“Deep Learning”) OR INDEXTERMS(“Supervised Machine Learning”) OR INDEXTERMS(“Unsupervised Machine Learning”) OR (TITLE-ABS(“Artificial Intelligence”) OR TITLE-ABS(AI) OR TITLE-ABS(“Machine Learning”) OR TITLE-ABS(“Deep Learning”) OR TITLE-ABS(“automated identification”) OR TITLE-ABS(“predictive model”) OR TITLE-ABS(“prediction model”) OR TITLE-ABS(“ensemble models”) OR TITLE-ABS(“ensemble learning”) OR TITLE-ABS(“ensemble methods”) OR TITLE-ABS(“risk prediction”)))

AND

(INDEXTERMS(“General Surgery”) OR INDEXTERMS(“Surgical Procedures, Operative”) OR INDEXTERMS(“Specialties, Surgical”) OR INDEXTERMS(“Early Detection of Cancer”) OR INDEXTERMS(“Neoplasm Metastasis”) OR (TITLE-ABS(Surgery) OR TITLE-ABS(Surgical) OR TITLE-ABS(“Cancer screening*”) OR TITLE-ABS(“Cancer diagnos*”) OR TITLE-ABS(Malignant) OR TITLE-ABS(Metastasis)))

AND

(INDEXTERMS(“Education, Medical, Graduate”) OR INDEXTERMS(“Internship and Residency”) OR (TITLE-ABS(Clinician*) OR TITLE-ABS(Physician*) OR TITLE-ABS(“Graduate Medical Education”) OR TITLE-ABS(“surgery resident*”) OR TITLE-ABS(“surgery residency*”) OR TITLE-ABS(“surgical residency*”) OR TITLE-ABS(“surgical resident*”) OR TITLE-ABS(“Surgical Trainee*”) OR TITLE-ABS(“Surgery Trainee*”) OR TITLE-ABS(“Surgical Training*”) OR TITLE-ABS(“Surgery Training*”) OR TITLE-ABS(“Medical Training*”) OR TITLE-ABS(“Residency Training*”) OR TITLE-ABS(“surgical education”) OR TITLE-ABS(“surgery education”)))

AND

(INDEXTERMS(“Data Accuracy”) OR INDEXTERMS(Prognosis) OR INDEXTERMS(Diagnosis) OR INDEXTERMS(“Early Diagnosis”) OR INDEXTERMS(“Risk Assessment”) OR (TITLE-ABS(“survival outcomes”) OR TITLE-ABS(accuracy) OR TITLE-ABS(Prognosis) OR TITLE-ABS(“Prognostic Accuracy”) OR TITLE-ABS(Diagnosing) OR TITLE-ABS(Diagnosis) OR TITLE-ABS(“diagnostic accuracy”) OR TITLE-ABS(“Risk Assessment”)))
